# Dynamic Changes of Endogenous Hormones in Different Seasons of *Idesia polycarpa* Maxim

**DOI:** 10.3390/life13030788

**Published:** 2023-03-15

**Authors:** Song Huang, Wei Zheng, Yanmei Wang, Huiping Yan, Chenbo Zhou, Tianxiao Ma

**Affiliations:** 1 College of Forestry, Xinyang Agricultural and Forestry University, Xinyang 464000, China; 2 College of Forestry, Henan Agricultural University, Zhengzhou 450002, China

**Keywords:** *Idesia polycarpa*, abscisic acid (ABA), indole-3-acetic acid (IAA), gibberellinA3 (GA3), trans-Zeatin-riboside (tZR), High-Performance Liquid Chromatography (HPLC)

## Abstract

*Idesia polycarpa* Maxim is a native dioecious tree from East Asia cultivated for its fruits and as an ornamental plant throughout temperate regions. Given the economic potential, comparative studies on cultivated genotypes are of current interest. This study aims to discover the dynamic changes and potential functions of endogenous hormones in *I. polycarpa*, as well as the differences in endogenous hormone contents in different growth stages among different *I*. *polycarpa* provenances. We used High-Performance Liquid Chromatography (HPLC) to measure and compare the levels of abscisic acid (ABA), indole-3-acetic acid (IAA), gibberellin A3 (GA3), and trans-Zeatin-riboside (tZR) in the leaves, flowers, and fruits of *I. polycarpa* from various provenances between April and October. Our findings indicated that changes in the ABA and GA3 content of plants from Jiyuan and Tokyo were minimal from April to October. However, the levels of these two hormones in Chengdu plants vary greatly at different stages of development. The peak of IAA content in the three plant materials occurred primarily during the early fruit stage and the fruit expansion stage. The concentration of tZR in the three plant materials varies greatly. Furthermore, we discovered that the contents of endogenous hormones in *I*. *polycarpa* leaves, flowers, and fruits from Chengdu provenances were slightly higher than those from Tokyo and Jiyuan provenances. The content of IAA was higher in male flowers than in female flowers, and the content of ABA, GA3, and tZR was higher in female flowers than in male flowers. According to the findings, the contents of these four endogenous hormones in *I. polycarpa* are primarily determined by the genetic characteristics of the trees and are less affected by cultivation conditions. The gender of *I. polycarpa* had a great influence on these four endogenous hormones. The findings of this study will provide a theoretical foundation and practical guidance for artificially regulating the flowering and fruiting of *I. polycarpa*.

## 1. Introduction

*Idesia polycarpa* Maxim is a dioecious tree in the Salicaceae family, which is economically significant and well-known throughout the temperate region [1,2]. There is only one species of this genus in the world [1]. This species is native to East Asia, including China, Japan, and Korea, and has since spread throughout the region [3]. There are obvious differences in the growth form, development speed, environmental adaptability, and physiological and biochemical characteristics of *I. polycarpa* due to long-term geographical isolation, natural selection, and climate [4].

*I. polycarpa* is a tall deciduous tree that blooms from April to May. It has yellow-green flowers with a fragrant scent. In the distribution area of *I*. *polycarpa*, the suitable annual average temperature ranges from 13 °C to 21 °C [5,6]. *I*. *polycarpa* is also known as the ‘Beautiful Tree Oil Depot’ because its fruits have a high oil content, in addition to being used as an ornamental and greening tree species [7]. The oil content in the flesh of the fruits ranges between 28.38 and 48.35%. In comparison, the oil content of the seeds is approximately 12.6–28.17%, and the oil extracted from the fruit has high commercial and medicinal value, with various applications, including edible oil, lubricating oil, biodiesel production, and medicines to reduce cholesterol and blood pressure [8]. Because *I. polycarpa* has an appealing color and shape and potential nutritional and medicinal value, more and more areas are attempting to cultivate it artificially. However, most *I. polycarpa* is still in the wild, and cultivation technology is not standardized or mature [9]. There are numerous factors that influence plant growth and development, but endogenous hormones, such as auxin (IAA), gibberellin (GA3), abscisic acid (ABA), and mitogen (tZR), play critical regulatory roles in the growth and development of *I. polycarpa* [10]. Currently, most *I. polycarpa* research focuses on ecology, cultivation, and breeding, with few studies on endogenous hormones. Endogenous hormone changes during flowering and fruiting, as well as their effects on the growth and development of *I. polycarpa*, remain unknown. Therefore, it is intended to investigate the dynamic changes in the endogenous hormones at various stages of the flowering and fruiting processes of *I. polycarpa.*

Plant hormones are a class of organic substances that are synthesized during plant metabolism and are required for various physiological processes in plants [11]. Plant growth regulators have been widely used in various developmental stages to improve plant growth and development, as well as the yield and quality of fruits, due to the importance of plant hormones to plant growth and regulation [12]. Previous research found that plant hormones directly affect fruit formation and development [13]. Therefore, it is essential to study the endogenous hormones of the sprouting, flowering, and fruiting of *I. polycarpa* from different origins and then apply exogenous hormones based on the local climate in the introduction and artificial planting process of this tree species to improve the success rate.

Auxin is a plant hormone that is essential for plant growth and development [14]. Auxin biosynthesis is important in a variety of plant development processes, including root development, embryogenesis, endosperm development, and flower development [15]. Similarly, IAA (indole-3-acetic acid) plays an important role in fruit formation and development [16]. Auxin response factor (ARF) controls the fate of fruit initiation events by controlling the level of gibberellin (GA) [17] and interacts with Aux/IAA proteins [18].

Abscisic acid (ABA) is a plant hormone derived from isoprene that accumulates as a result of a lack of water and is associated with seed dormancy, maturity, and development [19,20]. It also has a significant impact on plant responses to various abiotic stresses, such as drought, high temperature, low temperature, and salt [21]. ABA is also an important ripening control factor, as demonstrated by the following: (1) the content of ABA increased clearly in the early stages of apple fruit ripening [22]; (2) the content in peach and grapefruit fruits increases before ethylene release [23]; (3) exogenous ABA can promote the production of several metabolites related to fruit ripening [24]; (4) the fruits in tomato mutants lacking ABA could not maintain the normal growth pattern [25]; and (5) the maturation stage was delayed in ABA-deficient orange mutants [26].

Gibberellin (GA3) is a plant hormone that promotes cell division and proliferation [27]. GA3 is widely used in plant growth and development for a variety of purposes, including promoting seed germination and coping with abiotic stress [28], enhancing fruit growth [29], accelerating stem elongation [30], flowering [31], and other physiological effects caused by interactions with other plant hormones [32].

Trans zeatin riboside is a type of cytokinin that can promote cell division during tomato fruit development [33]. Liu et al. discovered that tZR has a significant impact on cucumber fruit growth and development [34]. Similarly, Honda et al. discovered that tZR plays an important role in pepper fruit expansion [35]. Furthermore, the content of tZR increased significantly during hop development, indicating that tZR was closely related to hop development [36].

It is well understood that phytohormones play an important role in seed germination. However, there is a lack of studies on how various endogenous hormones play a role in *I. polycarpa* and whether there are differences in the content of endogenous hormones in different growth stages of *I. polycarpa* from different provenances. The current study aims to quantify the changes in IAA, ABA, GA3, and tZR concentrations in the leaves, flowers, and fruits of *I. polycarpa* during different stages of development for three provenances from the natural range of the species. The findings provide a theoretical foundation for understanding the regulation of the flowering and fruiting period and the quantity of *I. polycarpa*, with potential applications in breeding, phenological manipulation, optimized cultivation, adequate harvesting activity planning, and performance evaluation.

## 2. Materials and Methods

### 2.1. Plant Materials and Growth Conditions

**Figure 1:**
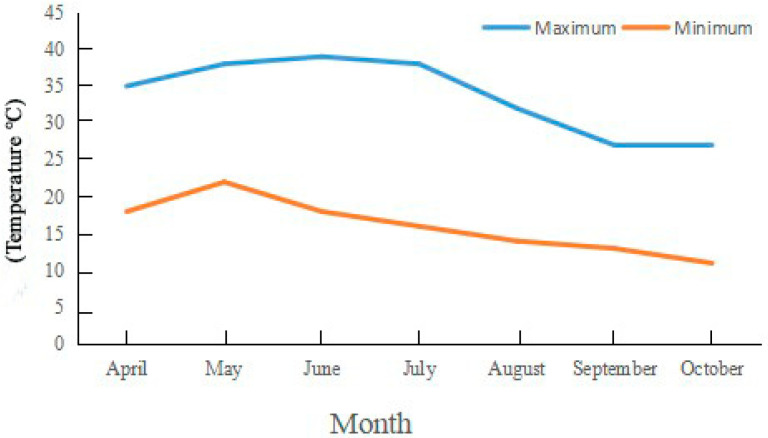
Monthly, minimum, and maximum temperature in Zhengzhou City, China. Note: The data come from https://www.tianqi24.com/zhengzhou/history2021.html (accessed on 2 January 2023).

Experiments were carried out at Henan Agricultural University’s Forestry Experiment Station in Zhengzhou City, China (113°38′ E, 34°48′ N). The research site is located in the warm temperate zone and has a temperate monsoon climate with an annual precipitation of ~650 mm. The maximum, minimum, and annual mean temperatures are 43.0 °C, −17.9 °C, and 14.2 °C, respectively, and the average annual sunshine duration is 2400 h (Figure 1, Table 1). The accumulated temperature ≥10 °C is 4717 °C, with a frost-free period of 215 d and a pH of about 7.0 in the experimental fields.

**Figure 2:**
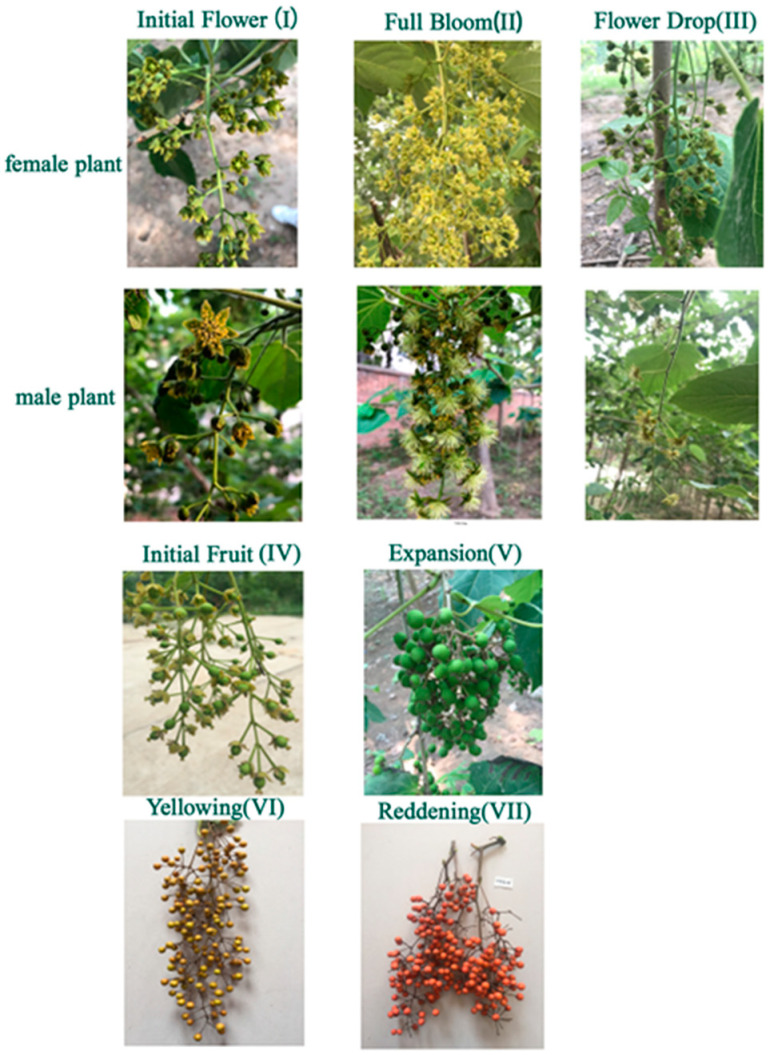
Morphological changes of *Idesia polycarpa* Maxim. During the flower stage and fruit stages.

The experimental material was *I. polycarpa* grown at Henan Agricultural University’s Forestry Experiment Station. Jiyuan (112°57′ E, 35°08′ N), Tokyo (139°69′ E, 35°68′ N), and Chengdu (104°07′ E, 30°67′ N) provided the plants. Tissue samples were collected from leaves, flowers, and fruits at various stages of development between April and October. The sampling period was divided into seven stages based on the stage of growth and development (Table 2, Figure 2). The plant samples were stored at −80 °C in liquid nitrogen. Furthermore, each sample was divided into three replicates.

### 2.2. Extraction and Analysis of Phytohormones

The concentrations of IAA, ABA, GA3, and tZR were determined using High-Performance Liquid Chromatography, which was slightly modified from Li [37]. We extracted the samples using a C_18_ solid phase extraction cartridge and analyzed them by HPLC-ESI-MS/MS using 0.05% formic acid in methanol and H_2_O as mobile phases for HPLC because the plant samples were different. Lyophilized samples were ground in liquid nitrogen, and 0.5 g powdered samples were extracted with 5 mL acetonitrile extraction solvent containing 30 µg/mL antioxidant. For 12 h, the extracted samples were refrigerated at 4 °C. After centrifuging the extract (10,000 rpm for 20 min at 4 °C), the supernatants were collected and re-extracted with 5 mL of extraction solvent before centrifuging the extraction solution (10,000 rpm for 10 min at 4 °C) again. The supernatants were then combined and distilled in a rotary evaporator at 40 °C before being dissolved in 4 mL of chloroform and 8 mL of phosphate buffer. The mixture was then treated with 150 mg PVPP (polyvinylpyrrolidone) and centrifuged at 8000 rpm for 10 min at 4 °C. The pH was adjusted to 3.0, and 3 mL of the mixture was treated with formic acid and extracted three times with an equal volume of ethyl acetate. The extracted samples were combined and distilled using a rotary evaporator (40 °C), after which they were redissolved in 1 mL of methanol and filtered through a strainer (0.22 µm). Finally, each sample was injected into the HPLC with 10 µL. The following were the mobile phase conditions: water—methanol (52:48) with 0.5‰ formic acid; column temperature—40 °C; detection wavelength—254 nm; injection volume—10 µL; flow rate—0.7 mL/min, constant gradient elution for 23 min.

#### Calculation Method of Endogenous Hormone Content

The standard curve was based on different peaks, and each peak time corresponds to different concentrations of calibration samples corresponding to plant hormones; the hormone content was calculated using the following method [38]:

Endogenous hormone content (µg·g^−1^) = As·V Css Vss/(Ass Ms·Vs)

As: Peak area of the sample; V: Volume of a final constant volume of sample pretreatment (mL); Css: Concentration of standard sample (g·L^−1^); Vss: Injection volume of standard sample (µL); Ass: Peak area of the standard sample; Ms: Dry weight of the sample (g); Vs: Injection volume of sample (mL).

### 2.3. Analytical Methods

Origin 2017 was used to create the graph for each hormone analysis. Using SPSS 24.0 software (IBM, Armonk, NY, USA), analysis of variance (ANOVA) and Duncan’s test at *p* = 0.05 or *p* = 0.01 were used to compare the variations of endogenous hormone levels in samples from different provenances and sample time treatments.

## 3. Results

### 3.1. Changes of Hormones in Female Leaves of I. polycarpa from Different Provenances

**Figure 3:**
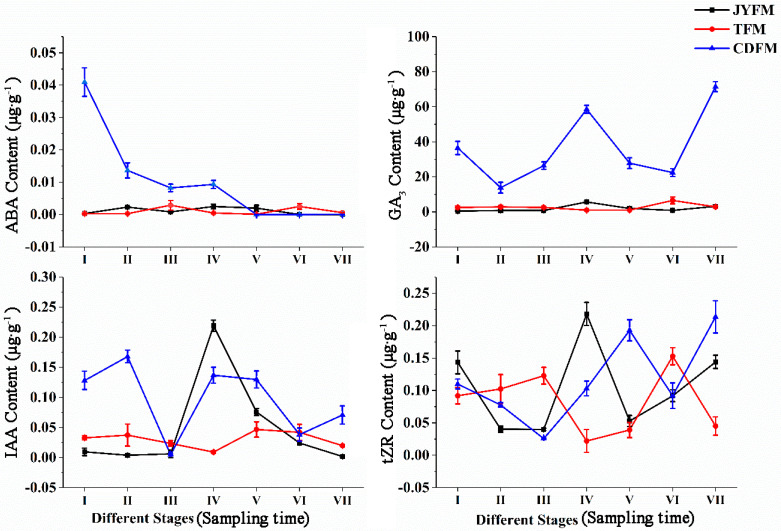
Dynamic changes of hormones in female leaves of *I. polycarpa* from April to October.

Endogenous concentrations of ABA, GA3, IAA, and tZR in female leaves of *I. polycarpa* from various provenances were examined (Figure 3). There were no discernible differences in the ABA content of plants from Jiyuan and Tokyo. On the contrary, the ABA content of Chengdu plants decreased rapidly, particularly from stage I to stage II. Because ABA biosynthesis is closely related to carotenoid synthesis, we infer that the decrease in ABA is related to the orange deepening of mature fruits. During the developmental stage I, the ABA content of plants from Chengdu was significantly higher than that of plants from the other two provenances (*p* < 0.01).

The change in trends of GA3 content was comparable to that of ABA within the Jiyuan and Tokyo plants. The difference is that the GA3 content of plants from Chengdu exhibited a “W” trend, which fluctuates significantly more than the other two provenances. GA3 levels were higher in stages IV (58.567 µg·g^−1^) and VII (71.450 µg·g^−1^), indicating that GA3 could promote fruit formation and development. In every stage of development, the ABA content of Chengdu plants was significantly higher than that of the other two provenances (*p* < 0.05).

The change in IAA content in Jiyuan provenance plants showed a rising and then falling trend. The content of IAA increased and then decreased with the typical double peak trend in the plants from Tokyo and Chengdu, but it fluctuated more pronouncedly in the Chengdu plants. The plants’ IAA content was highest in stages II, IV, and V. IAA was critical in regulating plant growth, particularly during the early stages of flowering and fruiting. Lower IAA concentrations were required for fruit ripening. During stages, I and II, the IAA content of Chengdu plants was significantly higher than that of the other two provenances (*p* < 0.05).

The overall findings revealed that there were significant differences in the content of tZR in plants from all three provenances. The highest concentration of tZR was found in stage IV (0.218 µg·g^−1^) for Jiyuan plants and stage VII (0.214 µg·g^−1^) for Chengdu plants. However, the level of tZR in the Tokyo plants was highest in stage VI, with a concentration of 0.153 µg·g^−1^. The highest concentration of tZR content was found in the first three stages of fruit development, indicating that tZR could promote cell division and was closely linked to fruit development. During stage V, the tZR content of Chengdu plants was higher than that of the other two provenances (*p* < 0.05).

### 3.2. Changes of Hormones in Male Leaves of I. polycarpa from Different Provenances

**Figure 4:**
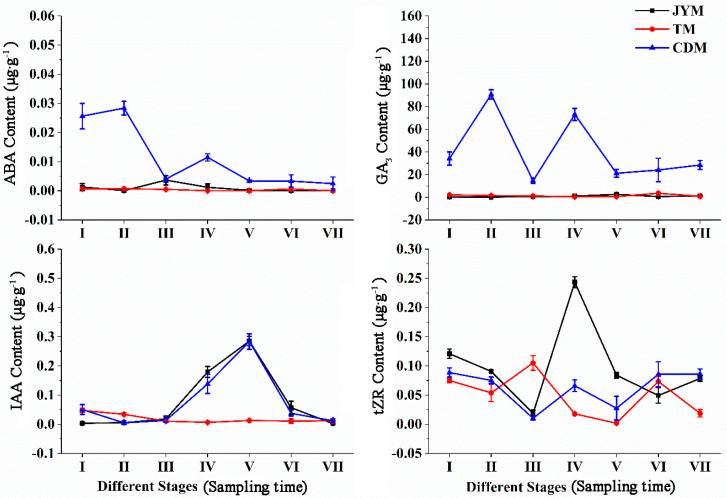
Dynamic changes of hormones in male leaves of *I. polycarpa* from April to October.

Figure 4 depicts the dynamic changes in hormones in male leaves of *I. polycarpa* from April to October. Male leaf ABA content changes were comparable to female leaf ABA content changes in Jiyuan and Tokyo. However, there was an unusual fluctuation in the ABA content of Chengdu plants from stage II to stage IV, as shown in the figure. The ABA content of Chengdu male leaves was significantly higher than that of the other two provenances in stages I, II, and IV (*p* < 0.01).

Changes in the GA3 content of female leaves were not visible in Jiyuan or Tokyo. The GA3 content in Chengdu leaves was significantly higher at all stages than in the other two provenances (*p* < 0.01), and the changing trend of the M-type was obvious.

The shift in IAA content was not immediately apparent from Tokyo. While the IAA content of male leaves from Jiyuan and Chengdu was highest at stage V, the content of other stages was comparable to Jiyuan. In stages IV and V, the IAA content from Tokyo was significantly lower than that from the other two provenances (*p* < 0.01).

The tZR content in Jiyuan male leaves was highest in stage IV (0.243 µg·g^−1^), with the greatest fluctuation. From stage I to stage VII, the content of tZR from Tokyo and Chengdu had a smaller change trend, with a periodic rise and fall. In stage V, the tZR content of male leaves from Jiyuan was significantly higher than that of the other two provenances (*p* < 0.01).

### 3.3. Changes of Hormones in Female Flowers of I. polycarpa from Different Provenances

**Figure 5:**
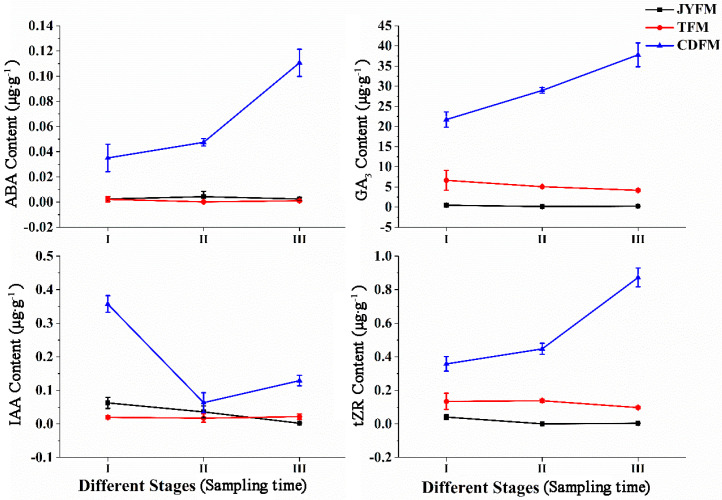
Dynamic changes of hormones in female flowers of *I. polycarpa* from April to May.

The levels of ABA, GA3, IAA, and tZR in female flowers differed statistically between *I. polycarpa* provenances (Figure 5). Changes in the contents of ABA, GA3, IAA, and tZR were not observed in female flowers from Jiyuan and Tokyo, but were observed in Chengdu flowers. The maximum values of ABA, GA3, IAA, and tZR were in stages III (0.111 µg·g^−1^), III (37.811 µg·g^−1^), I (0.357 µg·g^−1^), and III (0.873 µg·g^−1^), respectively. This indicated that IAA promotes flower opening and fruit formation.

The increasing ABA content in Chengdu flowers suggests that ABA is related to female flower abscission in the later period. Similarly, the increasing content of GA3 in Chengdu flowers suggests that GA3 is related to plant fruit setting. The increasing content of tZR in the same Chengdu flowers, on the other hand, suggests that tZR can promote female flower differentiation. In stages II and III, the content of ABA, GA3, and tZR in female flowers from Chengdu was significantly higher than in the other two provenances (*p* <0.05). Similarly, Chengdu’s IAA content in stage I was significantly higher than that of the other two provenances (*p* < 0.05).

### 3.4. Changes of Hormones in Male Flowers of I. polycarpa from Different Provenances

**Figure 6:**
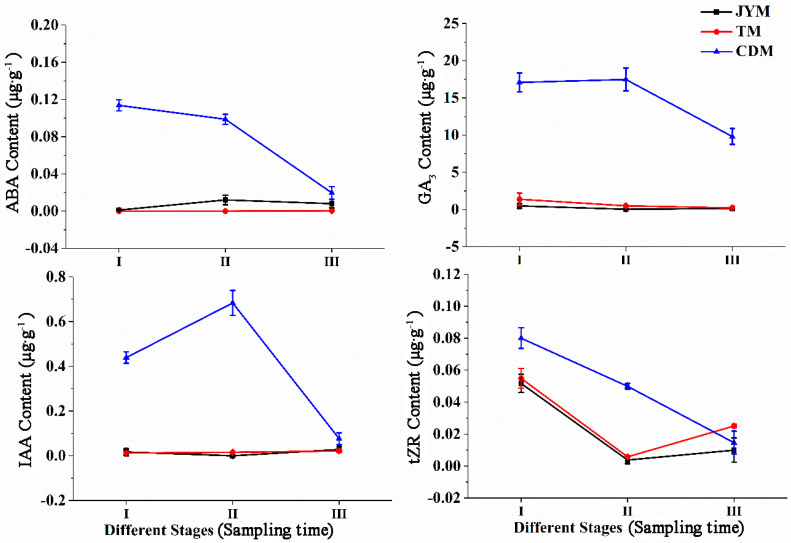
Dynamic changes of hormones in male flowers of *I. polycarpa* from April to May.

Changes in the content of ABA, GA3, and IAA were not observed in male flowers from Jiyuan and Tokyo, as shown in Figure 6, and the content of tZR decreased first and then increased. The ABA content of male flowers from Chengdu was higher in stages I (0.114 µg·g^−1^) and II (0.099 µg·g^−1^), but lower in stage III (0.020 µg·g^−1^), possibly due to ABA transfer from flowers to leaves. Chengdu flowers had significantly higher ABA content in stages I and II than in the other two provenances (*p* < 0.01). The primary function of ABA is to promote male flower differentiation and flowering.

GA3 levels in Chengdu male flowers were highest in stages I (17.081 µg·g^−1^) and II (17.476 µg·g^−1^). Furthermore, based on our findings, it was clear that the GA3 content in Chengdu flowers at all stages was significantly higher than that of the other two provenances (*p* < 0.01).

The IAA content of male flowers from the Jiyuan and Tokyo provenances was low throughout all stages; however, the IAA content of male flowers from Chengdu was highest in stage II (0.683 µg·g^−1^), indicating that IAA promotes male flower flowering at this stage of development. Furthermore, we discovered that the IAA content of Chengdu flowers in stages I and II was significantly higher than that of the other two provenances (*p* < 0.01).

The content of tZR revealed that its peak levels of flowers from Jiyuan, Tokyo, and Chengdu occurred in stage I, followed by a drop in stages II and III. This phenomenon demonstrated that tZR was associated with the formation of male flowers. Overall, the tZR content of the Chengdu flowers in stage II was significantly higher than that of the other two provenances (*p* < 0.01).

### 3.5. Changes of Hormones in Fruits of I. polycarpa from Different Provenances

**Figure 7:**
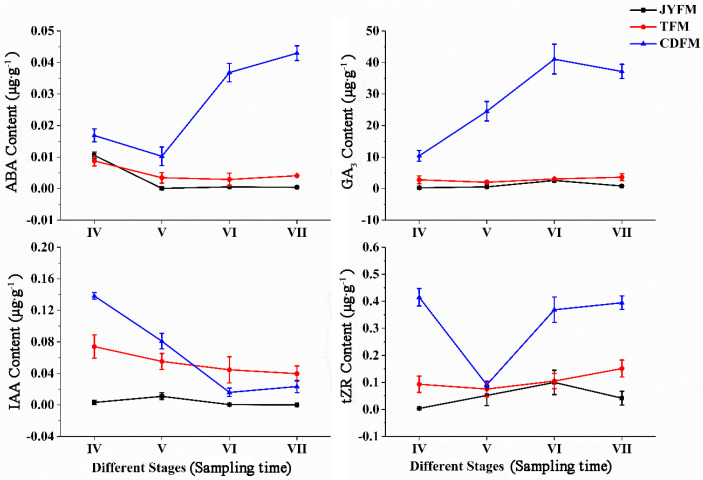
Dynamic changes of hormones in fruits of *I. polycarpa* from May to October.

Figure 7 depicts the changes in hormone concentrations. The concentration of ABA did not change significantly in fruits from Jiyuan and Tokyo, but it did fall and then rise in Chengdu fruits. The ABA content of Chengdu fruits in stages VI and VII was significantly higher than that of the other two provenances (*p* < 0.05). This phenomenon may be associated with the production of stress-resistant proteins in response to external stress.

The content of GA3 was unchanged in the fruits from Jiyuan and Tokyo, but increased gradually and then decreased in the fruits from Chengdu. The highest level of GA3 in the latter case was stage VI (0.043 µg·g^−1^). The GA3 content in Chengdu fruits in stages VI and VII was significantly higher than in the other two provenances (*p* < 0.05).

The content of IAA in the fruits from Jiyuan did not change significantly, while the content of IAA in the fruits from Tokyo and Chengdu had the highest peak values of 0.074 µg·g^−1^ and 0.138 µg·g^−1^ in stage IV, respectively. The lower IAA content in stage VI suggested that fruit ripening is more sensitive to IAA, whereas higher IAA content could have the opposite effect.

The content of tZR in the fruits from Tokyo and Chengdu had maximum values at stage VII (0.151 µg·g^−1^) and stage IV (0.415 µg·g^−1^), respectively. In the case of the fruits from Jiyuan, the maximum level was in stage VI, with a peak value of 0.100 µg·g^−1^. The tZR content of Chengdu fruits in stages IV, VI, and VII was significantly higher than that of the other two provenances (*p* < 0.05). The tZR content in the fruits from the three provenances was similar in stage V, indicating that a certain amount of tZR could promote fruit differentiation.

## 4. Discussion

The growth and development of *I. polycarpa* were the results of the combined action of many endogenous hormones, including ABA, GA3, IAA, and tZR, each of which had different effects on different organs and stages. The literature currently lacks information on whether cultivation conditions affect the four endogenous hormones in different organs and stages; thus, this study provides a research foundation for its cultivation and development.

Earlier research suggested that ABA could accelerate or delay the flowering time in different plant species and developmental stages. In this study, the ABA content of female flowers from Chengdu increased significantly during the falling flowers period. Many plant hormones, such as abscisic acid, ethylene, and jasmonic acid, are important in regulating organ aging and abscission mechanisms, according to Mohd Gulfishan [39]. In the study of the molecular mechanism of ABA-induced leaf senescence [40], it was also demonstrated that ABA can promote organ dormancy and abscission and has a role in modulating plant stress resistance. Furthermore, the ABA content of Chengdu provenance fruits increased over time. In woodland strawberries, ABA was found to play a coordinating role in fruit growth and ripening [41].

The content of GA3 increased in female flowers from Chengdu, but decreased in male flowers during the falling flowers period, indicating that GA3 was related to fruit setting and male flower differentiation. Watanabe et al. discovered that exogenous GA3 could promote apple fruit formation [42]. Gibberellin could activate and maintain cell division of the ovary wall in citrus, leading to fruit setting [43]. Other research has found that GA3 can promote vegetative growth and flower bud differentiation [44]. From the initial fruit period to the fruit yellowing period, the content of GA3 in Chengdu fruits has increased. Gibberellin was discovered to promote parthenocarpy and fruit expansion in sweet cherries, as evidenced by the qPCR results of related genes, the fruit setting rate, and the parthenocarpy fruit size [45]. More interestingly, high temperature promotes the content of endogenous hormones for seed germination in *I*. *polycarpa*, causing the balance of endogenous hormone content to break and the ratio of GA3/ABA to increase toward seed germination. Our data showed that the content of GA3 and ABA in the fruit showed an inverse trend and that in the later fruit, both were increased, implying that a balance between them was achieved in the fruit.

The initial flowering stage and full flowering stage of Chengdu flowers had higher IAA content. Physiological and molecular research on *Arabidopsis thaliana* revealed that polar auxin transport was required for flower formation [46]. High-performance liquid chromatography (HPLC) [47] was used to investigate the relationship between IAA and flower formation, and it was discovered that high levels of IAA promoted flower bud induction in apple trees (*Malus pumila*), whereas Liu et al. discovered that low levels of IAA promote flowering in loquat (*Eriobotrya japonica*) [48]. Furthermore, endogenous auxin concentration was the limiting factor in controlling apple fruit size [49]. Almudena Bermejo discovered that IAA can regulate GA metabolism in citrus, resulting in significant changes in the level of active GA1 in the ovule and pericarp and, ultimately, fruit setting [50]. Increased ethylene production, which directly regulates fruit ripening, necessitates higher IAA concentrations for fruit ripening [51]. Our research also revealed that the IAA content in the fruits of the three provenances was higher before the fruit turned yellow, but then dropped until the fruit was fully ripe.

The tZR content of Jiyuan flowers was higher during the early flowering stage. tZR typically promotes female flower development, as evidenced by the higher tZR content in female flowers from the initial flowering stage to the falling flowers stage in a study on *Glycyrrhiza uralensis* [52]. Male flowers had a relatively high tZR content at the start of flower formation, which is supported by the fact that high IAA and tZR contents during the flower bud differentiation stage are conducive to flower bud differentiation [53]. tZR also inhibited many developmental processes, such as bud and root elongation, cell differentiation, bud regeneration, and meristem activity [54,55].

Previous research has shown that high IAA and tZR contents promote flower bud differentiation, whereas high GA and ABA contents promote flower and fruit abscission [52]. High levels of IAA and tZR regulate plant vegetative growth and flower induction [56]. Furthermore, ABA promotes flowering, whereas GA3 and IAA do not [57]. A similar study on the changes in endogenous hormones during the flowering period of *Gnetum parvifolium* found that high levels of GA3 and tZR promote male flower differentiation, while high levels of IAA promote female flower differentiation [58]. The peak contents of different plant organs’ endogenous hormones may be related to the transfer of such hormones to different organs at different stages.

Plants can change their growth and development in response to their surroundings, which is controlled by endogenous plant hormones. Several endogenous hormones, including ABA, GA3, IAA, and tZR, act together to promote the growth and development of *I*. *polycarpa*. Each hormone had a distinct effect on different organs and developmental stages. The fluctuation was obvious, and the concentrations of several endogenous hormones in various organs of the Chengdu provenance were significantly higher than those of the other two provenances. Geographical location and environmental factors, such as light intensity/availability, soil quality, and precipitation, may all have a causal relationship with endogenous hormone concentration variation. Nonetheless, these environmental influences have a minor impact. According to the findings, the contents of these four endogenous hormones in *I. polycarpa* are primarily determined by genetic characteristics of the trees and are less affected by cultivation conditions. The gender in *I. polycarpa* had a great influence on these four endogenous hormones.

## 5. Conclusions

The materials used in the experiment came from the Forestry Experimental Station of Henan Agricultural University. The differences in hormone contents between provenances could be due to genetic differences in *I. polycarpa*. The contents of various endogenous hormones in different organs of the Chengdu provenance were significantly higher than those of the other two provenances, with a clear fluctuation. This discovery may provide insight into how differences in endogenous hormone concentration can lead to differences in flowering and fruiting time and quantity, providing cultural direction. In the future, we will increase the sample size and lengthen the period of observation to confirm the results.

## Figures and Tables

**Table 1:** Monthly statistics of air quality in the experimental area.

Month	NO_2_	CO	SO_2_	O_3_
2021–04	31.3 ± 1.53	0.64 ± 0.08	9.17 ± 1.75	113.5 ± 2.36
2021–05	25.9 ± 3.06	0.61 ± 0.13	9.42 ± 2.16	143.8 ± 3.78
2021–06	23.7 ± 3.51	0.71 ± 0.19	6.81 ± 2.39	178.6 ± 4.31
2021–07	16.3 ± 1.27	0.56 ± 0.11	3.40 ± 0.86	134.3 ± 4.54
2021–08	17.6 ± 2.55	0.66 ± 0.24	4.47 ± 1.59	137.9 ± 3.21
2021–09	24.1 ± 4.51	0.75 ± 0.16	5.93 ± 2.13	126.4 ± 7.52
2021–10	42.9 ± 3.72	0.81 ± 0.21	8.74 ± 2.28	84.1 ± 4.14

Note: The data came from the Henan Meteorological Service.

**Table 2:** Sampling Time of *I. polycarpa*.

Period	Stage		Sample Time		
			Jiyuan	Tokyo	Chengdu
Initial Flower	Early April–Late April	I	24 April 2021	26 April 2021	26 April 2021
Full Bloom	Late April–Early May	II	28 April 2021	1 May 2021	1 May 2021
Flower Drop	Early May	III	4 May 2021	6 May 2021	6 May 2021
Initial Fruit	Early May–Late May	IV	13 May 2021	16 May 2021	16 May 2021
Expansion	Late May–Middle August	V	24 May 2021	26 May 2021	26 May 2021
Yellowing	Middle August–Early September	VI	1 September 2021	3 September 2021	3 September 2021
Reddening	Early September–Early October	VII	8 October 2021	10 October 2021	10 October 2021

## Data Availability

The data presented in this study are available on request from the corresponding author.
